# Gadoxetic acid-enhanced magnetic resonance imaging predicts hyperbilirubinemia induced by glecaprevir during hepatitis C virus treatment

**DOI:** 10.1038/s41598-022-11707-6

**Published:** 2022-05-12

**Authors:** Hironao Okubo, Masanori Atsukawa, Tomomi Okubo, Hitoshi Ando, Eisuke Nakadera, Kenichi Ikejima, Akihito Nagahara

**Affiliations:** 1 Department of Gastroenterology, Juntendo University Nerima Hospital, 3-1-10 Takanodai, Nerima-ku, Tokyo, 177-8521 Japan; 2 Division of Gastroenterology, Department of Internal Medicine, Nippon Medical School Chiba Hokusoh Hospital, 1715 Kamagari, Inzai, Chiba 270-1694 Japan; 3 Division of Gastroenterology, Department of Internal Medicine, Nippon Medical School, 1-1-5 Sendagi, Bunkyo-ku, Tokyo, 113-8603 Japan; 4 Department of Cellular and Molecular Function Analysis, Graduate School of Medical Sciences, Kanazawa University, 13-1 Takara-machi, Kanazawa, 920-8640 Japan; 5 Department of Gastroenterology, Graduate School of Medicine, Juntendo University, 3-1-3 Hongo, Bunkyo-ku, Tokyo, 113-8421 Japan

**Keywords:** Diseases, Gastroenterology, Medical research

## Abstract

Glecaprevir is a substrate for organic anion-transporting polypeptide (OATP) 1B1/1B3, which transports bilirubin. Hyperbilirubinemia is an adverse event during anti-hepatitis C virus treatment with glecaprevir and pibrentasvir. Gadoxetic acid is also transported by OATP1B1/1B3, and we aimed to evaluate whether gadoxetic acid-enhanced magnetic resonance (MR) imaging was associated with glecaprevir trough concentrations (C_trough_). We further determined whether this was predictive of hyperbilirubinemia development in a cohort of 33 patients. The contrast enhancement index (CEI), a measure of hepatic enhancement effect on the hepatobiliary image, was assessed. Glecaprevir C_trough_ was determined 7 days after administration. Five of the 33 patients (15%) developed Common Terminology Criteria for Adverse Events grade ≥ 2 hyperbilirubinemia. We found a negative relationship between CEI and C_trough_ (r = − 0.726, *p* < 0.001). The partial correlation coefficient between CEI and C_trough_ was − 0.654 (*p* < 0.001), while excluding the effects of albumin, FIB-4 index, and indirect bilirubin at baseline. The C_trough_ was significantly higher in patients with hyperbilirubinemia than in those without (*p* = 0.008). In multivariate analysis, CEI ≤ 1.71 was an independent factor influencing the development of hyperbilirubinemia (*p* = 0.046). Our findings indicate that gadoxetic acid MR imaging can help predict glecaprevir concentration and development of hyperbilirubinemia.

## Introduction

Recently, interferon-free direct acting antiviral (DAA) treatment has become the standard therapy for chronic hepatitis C virus (HCV) infection, achieving high serological viral responses^1,2^. Glecaprevir/pibrentasvir, a pangenotypic regimen, has been available in Japan since 2017^3^. Glecaprevir, a nonstructural (NS) 3/4 A protease inhibitor, is combined with pibrentasvir, a NS5A inhibitor. These drugs are predominantly eliminated via hepatobiliary excretion with minimal renal elimination^4^. Glecaprevir is a substrate and inhibitor for organic anion transporting-polypeptide (OATP) 1B1 and 1B3, which are hepatic uptake transporters that also carry unconjugated bilirubin^5–7^, and for efflux transporters breast cancer resistance protein (BCRP) and P-glycoprotein (P-gp). In contrast, pibrentasvir is a substrate and an inhibitor of both P-gp and BCRP^7^.

During hepatitis C treatment with the glecaprevir/pibrentasvir regimen, an elevation of serum bilirubin concentration, related to the inhibition of bilirubin transporters, has been observed in some cases^7,8^. Potential drug-bilirubin interaction, one type of adverse event during therapy, can occur with NS 3/4A protease inhibitors such as glecaprevir, paritaprevir, and simeprevir^9–11^. Adverse drug reaction remains an important aspect of management in DAA therapy. Clinically, in patients receiving glecaprevir/pibrentasvir, 5.3% had elevated total bilirubin levels of 1.5–3 times the upper limit of normal (ULN), equivalent to Common Terminology Criteria for Adverse Events (CTCAE) grade 2 while 0.5% demonstrated > 3 × ULN, equivalent to CTCAE grade ≥ 3^12^. Glecaprevir-induced hyperbilirubinemia is also presumably caused by the inhibitory effect of the drug on OATP1B1/1B3^13^. Nevertheless, there are few reports describing these interactions in clinical practice, or the relationship between the plasma concentration of glecaprevir and the development of hyperbilirubinemia.

Gadoxetic acid (gadolinium-ethoxybenzyl-diethylenetriamine pentaacetic acid), a liver-specific contrast agent for magnetic resonance (MR) imaging, is also a substrate of OATP1B1 and OATP1B3^14^. This background prompted us to undertake the present study to determine the relationship between hepatic enhancement by gadoxetic acid and both plasma glecaprevir concentration and bilirubin elevation in patients with hepatitis C who had undergone treatment with the glecaprevir/pibrentasvir regimen.

## Results

### Baseline characteristics and bilirubin increase during therapy

Table 1 summarizes the baseline characteristics of all patients included in this study. Their baseline median total bilirubin concentration was 0.6 mg/dL (range 0.3–1.4 mg/dL).

**Table 1:** Patients’ characteristics.

Characteristic	n = 33
Age (years)ª	60 (22–91)
Male/female	15/18
Past history of DAAs yes/no	3/30
Chronic hepatitis/liver cirrhosis	28/5
Aspartate aminotransferase (IU/L)ª	35 (16–171)
Alanine aminotransferase (IU/L)ª	44 (8–231)
Total bilirubin (mg/dL)ª	0.6 (0.3–1.4)
Direct bilirubin (mg/dL)ª	0.2 (0.1–0.5)
Indirect bilirubin (mg/dL)ª	0.4 (0.2–1.0)
Albumin (g/dL)ª	4.3 (3.3–4.9)
Alkaline phosphatase, IU/L	253 (130–931)
Prothrombin activity (%)ª	100 (76–144)
Platelet count (10^8^/L)ª	20.9 (9.4–36.9)
eGFR (mL/dL/1.13 m^2^)ª	76 (45–103)
Child–Pugh score, 5/6	29/4
FIB-4 indexª	1.92 (0.11–8.16)

*eGFR* estimated glomerular filtration rate, *DAA* direct acting antivirals, *FIB-4* fibrosis-4. ^a^Data are shown as median (range) values.

Of these 33 patients, five (15.2%) had liver cirrhosis. The ULN of the total bilirubin value at our institutions was 1.2 mg/dL. At baseline, 32 patients demonstrated CTCAE grade 0 for serum bilirubin, and one patient showed grade 1. Maximum total bilirubin during anti-viral therapy was 25 patients in grade 0, three patients in grade 1, and five patients in grade 2.

**Figure 1:**
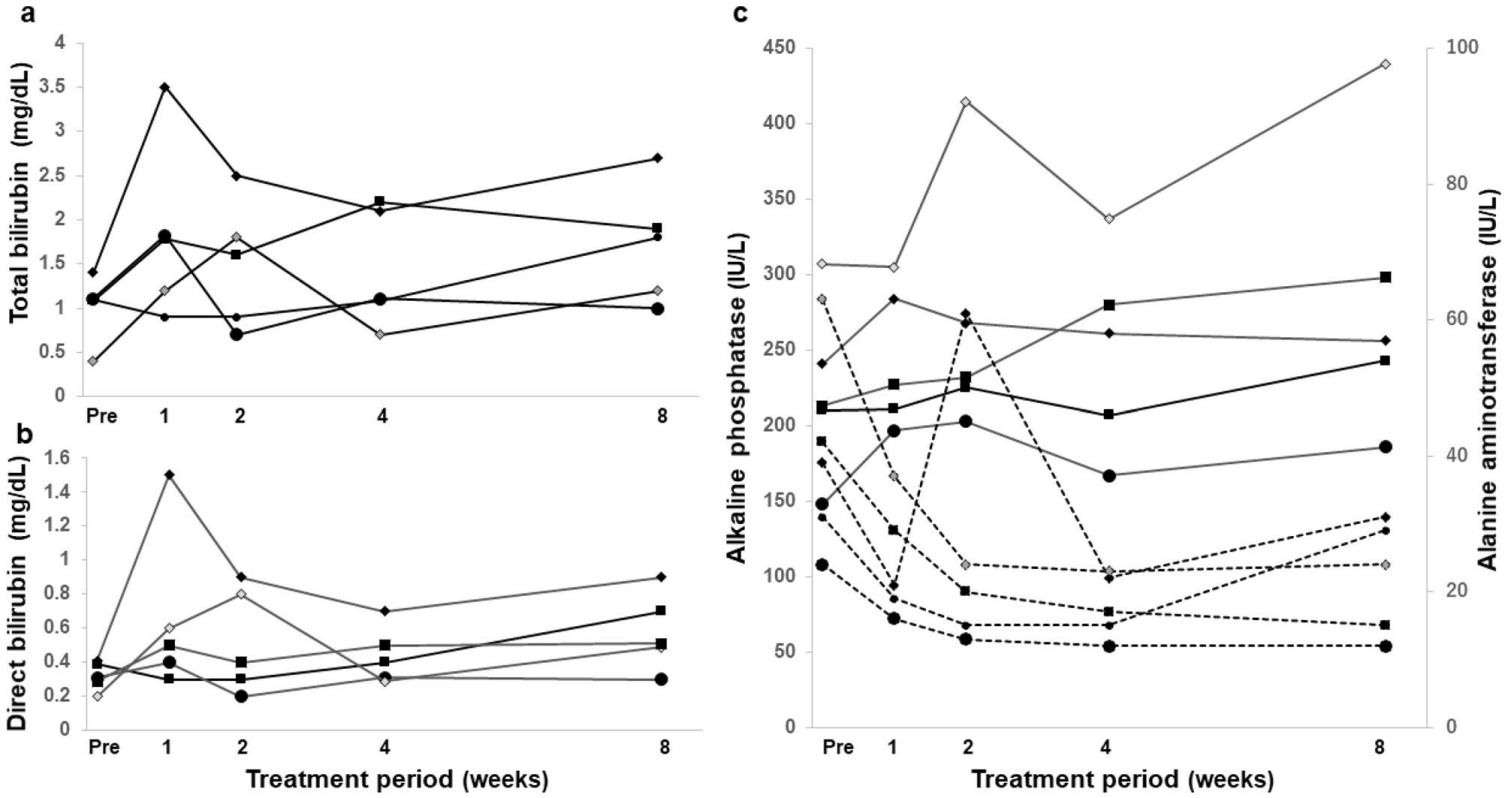
Time course of (**a**) total and (**b**) direct bilirubin concentration and (**c**) alanine aminotransferase and alkaline phosphatase in five patients treated with glecaprevir who developed grade ≥ 2 hyperbilirubinemia. Black line, total and direct bilirubin and alkaline phosphatase; dotted line, alanine aminotransferase.

Figure 1 shows the time course of bilirubin concentration in patients who developed grade ≥ 2 hyperbilirubinemia. During treatment, five patients (15.2%) with chronic hepatitis but without cirrhosis, had total bilirubin concentrations ≥ 1.8 mg/dL, equivalent to CTCAE grade ≥ 2. Of these five patients, one had a maximum total bilirubin concentration of 3.5 mg/dL, followed by 2.2, 1.8, 1.8, and 1.8 mg/dL in the remaining patients, respectively. Their indirect (unconjugated) bilirubin concentrations were 2.0, 1.7, 1.2, 1.1, and 1.0 mg/dL, respectively. Median onset time of maximum total bilirubin was 2 weeks (range 1–8 week) after the start of treatment. In the case of the patient with total bilirubin concentration of 3.5 mg/dL, the drug dose was reduced due to the accompanied elevation of grade 1 serum aminotransferase. The other four patients did not demonstrate concurrent elevation of serum aminotransferase.

### Relationships between plasma concentration of glecaprevir and variables including MR imaging analysis

**Figure 2:**
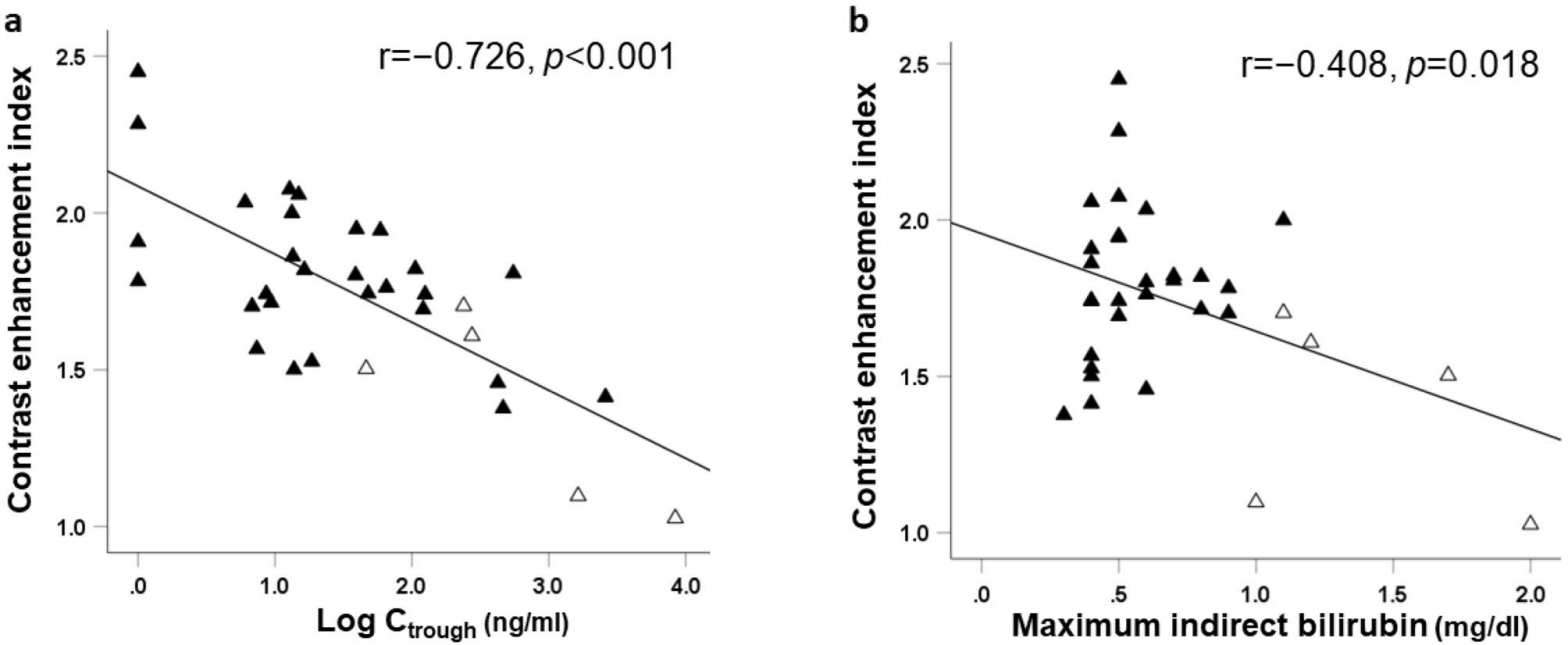
Relationship between contrast enhancement index and (**a**) glecaprevir trough concentration, or (**b**) maximum indirect bilirubin. Black triangle, patients without grade ≥ 2 hyperbilirubinemia; open triangle, patients with grade ≥ 2 hyperbilirubinemia. *CEI* contrast enhancement index, *C*_*trough*_ trough concentration of glecaprevir. Data were analyzed using Pearson’s correlations.

Figure 2 illustrates the relationship between contrast enhancement index (CEI) and Log_10_ C_trough_ of glecaprevir or maximum indirect bilirubin concentration. The Log_10_ C_trough_ showed significant negative correlations with CEI (r = − 0.726, *p* < 0.001) (Fig. 2a). In addition, there was a negative correlation between CEI and maximum indirect bilirubin concentration (r = − 0.408; *p* = 0.018) (Fig. 2b). Furthermore, the Log_10_ C_trough_ showed significant correlations with maximum indirect bilirubin concentration (r = 0.754, *p* = 0.043).

Correlation coefficients and partial correlation coefficients between Log_10_ C_trough_ of glecaprevir and variables such as serum albumin, FIB-4 index, and indirect bilirubin at baseline are presented in Table 2. CEI was significantly correlated with serum albumin (r = 0.417; *p* = 0.016), while Log_10_ C_trough_ showed significant negative correlation with serum albumin (r = − 0.346; *p* = 0.049). However, serum indirect bilirubin concentration at baseline did not significantly correlate with Log_10_ C_trough_ or CEI (r = 0.262; *p* = 0.141, r = − 0.203; p = 0.258, respectively). The partial correlation coefficient between CEI and Log_10_ C_trough_ was − 0.654 (*p* < 0.001), while excluding the effects of albumin, FIB-4 index, and indirect bilirubin at baseline.

**Table 2:** Correlation coefficients and partial correlation coefficients between variables.

	ALB	FIB-4 index	CEI	Log_10_ C_trough_	ID-Bil
ALB	–	− 0.387*	0.417*	− 0.346*	**− 0.043**
FIB-4 index	− 0.298	–	**− 0.296**	**0.233**	**− 0.151**
CEI	0.208	− 0.124	–	− 0.726**	**− 0.203**
Log_10_ C_troough_	− 0.060	0.048	− 0.654**	–	**0.262**
ID-Bil	− 0.012	− 0.227	− 0.060	0.181	–

The upper right of the table shows correlation coefficients in bold font, and the lower left shows partial correlation coefficients in normal font. *ALB* albumin, *CEI* contrast enhancement index, *FIB-4* fibrosis-4, *ID-Bil* indirect bilirubin, *C*_*trough*_ trough concentration of glecaprevir. Asterisks indicate statistically significant differences (*p < 0.05, **p < 0.001).

### Comparison of variable factors in patients with and without grade ≥ 2 hyperbilirubinemia

As shown in Table 3, the C_trough_ of glecaprevir was significantly higher in patients with hyperbilirubinemia (*p* = 0.008) compared with those without hyperbilirubinemia. In terms of pretreatment parameters, the CEI was significantly lower in patients with hyperbilirubinemia (*p* = 0.009) compared with those without. The values of serum total and indirect bilirubin were significantly higher in patients with hyperbilirubinemia than those without (*p* = 0.030 and *p* = 0.029, respectively). In contrast, there were no significant differences between these two groups in direct bilirubin (*p* = 0.183), serum albumin level (*p* = 0.860), presence of compensated cirrhosis (*p* = 0.569) and FIB-4 index (*p* = 0.482).

**Table 3:** Variables in patients with and without grade ≥ 2 hyperbilirubinemia before and during treatment.

Variable	With hyperbilirubinemia (n = 5)	Without hyperbilirubinemia (n = 28)	*p* value
**Before treatment**			
Age (years)^a^	62 (46–81)	60 (22–91)	0.960
Male/female	1/4	14/14	0.346
Body weight (kg)^a^	60.0 (38.5–80.0)	50.0 (45.0–88.0)	0.228
CH/LC	5/0	23/5	0.569
AST (IU/L)^a^	38 (26–55)	34 (16–171)	0.900
ALT (IU/L)^a^	62 (46–81)	62 (46–81)	0.514
Total bilirubin (mg/dL)^a^	1.1 (0.4–1.4)	0.6 (0.3–1.1)	0.030
Direct bilirubin (mg/dL)^a^	0.3 (0.2–0.4)	0.2 (0.1–0.5)	0.183
Indirect bilirubin (mg/dL)^a^	0.8 (0.2–1.0)	0.4 (0.2–0.7)	0.029
Albumin (g/dL)^a^	4.2 (3.8–4.5)	4.3 (3.3–4.9)	0.860
ALP (IU/L)^a^	213 (148–307)	260 (130–931)	0.132
Prothrombin activity (%)^a^	105 (76–140)	99.5 (76–140)	0.547
Platelet count (× 10^8^/L)^a^	24.3 (13.3–34.3)	20.75 (9.4–36.9)	0.353
eGFR (mL/dL/1.13 m^2^)^a^	63.6 (56.5–78.1)	76.7 (45.0–103.0)	0.120
FIB-4 index^a^	1.35 (1.08–4.31)	1.97 (0.11–8.16)	0.482
CEI^a^	1.503 (1.03–1.70)	1.792 (1.38–2.45)	0.009
**During treatment**			
Log C_trough_ (ng/mL)^a^	2.439 (1.67–3.92)	1.194 (0–3.41)	0.008

*AST* aspartate aminotransferase, *ALT* alanine aminotransferase, *ALP* alkaline phosphatase, *eGFR* estimated glomerular filtration rate, *FIB-4* fibrosis-4, *CH/LC* chronic hepatitis/liver cirrhosis, *CEI* contrast enhancement index, *C*_*trough*_ trough concentration of glecaprevir. ^a^Data are shown as median (range) values.

### ROC curve analysis

As listed in Table 4, receiver operating characteristic (ROC) curve analysis revealed that total bilirubin ≥ 0.8 mg/dL and indirect bilirubin ≥ 0.5 mg/dL at baseline, and CEI ≤ 1.71 were the best cutoff values to predict the development of grade ≥ 2 hyperbilirubinemia. Specifically, the CEI was highly accurate in predicting hyperbilirubinemia (AUROC 0.871, 95% CI 0.737–1.000, *p* = 0.009, sensitivity 100%, specificity 71.4%, positive predictive value 38.5% and negative predictive value 100%).

**Table 4:** ROC curve analysis of each baseline parameter and prediction of grade ≥ 2 hyperbilirubinemia.

	Cut-off value	AUROC (95% CI)	*p* value	Sensitivity, %	Specificity, %	PPV, %	NPV, %
Total bilirubin (mg/dL)	0.8	0.804 (0.480–1.000)	0.033	80.0	78.5	40.0	95.7
Indirect bilirubin (mg/dL)	0.5	0.804 (0.473–1.000)	0.033	80.0	92.9	66.7	96.3
CEI	1.71	0.871 (0.737–1.000)	0.009	100.0	71.4	38.5	100.0

*AUROC* area under the receiver operating characteristic curve, *CEI* contrast enhancement index, *CI* confidence interval, *NPV* negative predictive value, *PPV* positive predictive value.

### Multivariate analysis factors associated with development of grade ≥ 2 hyperbilirubinemia

As shown in Table 5, univariate and multivariate logistic regression analyses of baseline factors related to the development of grade ≥ 2 hyperbilirubinemia were performed. Univariate logistic regression analysis revealed that indirect bilirubin ≥ 0.5 mg/dL, estimated glomerular filtration rate ≤ 60 mL/dL/1.13 m^2^, and CEI ≤ 1.71 tended to relate to the development of hyperbilirubinemia (*p* = 0.073, *p* = 0.065, and *p* = 0.054, respectively). By multiple logistic regression analysis, CEI was a significant and independent predictor associated with developing grade ≥ 2 hyperbilirubinemia (odds ratio 13.781, 95% CI 1.045–181.772; *p* = 0.046).

**Table 5:** Univariate and multivariate logistic regression analyses of baseline factors related to the development of grade ≥ 2 hyperbilirubinemia during glecaprevir/pibrentasvir treatment.

Variable	Univariate analysis			Multivariate analysis		
	OR	95% CI	*p* value	OR	95% CI	*p* value
Age, ≥ 75 years	2.000	0.275–14.531	0.493			
Sex, male	0.250	0.025–2.527	0.240			
Body weight, ≥ 60 kg	0.217	0.021–2.191	0.195			
Chronic hepatitis, yes	3.512 × 10^8^	0.0–NA	0.999			
Indirect bilirubin, ≥ 0.5 mg/dL	8.444	0.821–86.828	0.073	11.849	0.889–157.918	0.061
Albumin, ≥ 4.0 g/dL	1.600	0.154–16.605	0.694			
Prothrombin activity, ≥ 80%	0.480	0.040–5.831	0.565			
Platelet count, ≥ 20 × 10^8^/L	0.578	0.083–4.009	0.579			
eGFR, ≤ 60 mL/dL/1.13 m^2^	8.667	0.873–86.062	0.065			
FIB-4 index, ≥ 2.67	0.288	0.029–2.917	0.292			
CEI, ≤ 1.71	10.00	0.964–103.779	0.054	13.781	1.045–181.772	0.046

*CI* confidence interval, *CEI* contrast enhancement index, *eGFR* estimated glomerular filtration rate, *FIB-4* fibrosis-4, *NA* not applicable, *OR* odds ratio.

## Discussion

In this study, we have shown that liver parenchymal enhancement effect at 20 min of gadoxetic acid-enhanced MR imaging was independently predictive of glecaprevir C_trough_ and the development of grade ≥ 2 hyperbilirubinemia in patients with HCV treated with the glecaprevir/pibrentasvir regimen. We also found that the enhancement effect correlated with maximum indirect bilirubin, which indicates the severity of hyperbilirubinemia.

Chronic liver disease is related to reduced capacity of drug metabolic enzymes, but it may also affect a wide array of pharmacological factors including expression or function of drug transporters, hepatic blood flow, and plasma protein binding capacity^7,15^. In the EASL guideline, treatment regimens comprising an NS3/4A protease inhibitors, such as paritaprevir, grazoprevir, glecaprevir or voxilaprevir, must not be used in patients with Child–Pugh B or C decompensated cirrhosis, due to the remarkable elevation of protease inhibitor concentrations and the related risk of toxicity^16^. Our findings suggest that the development of hyperbilirubinemia was associated with high plasma concentration of glecaprevir. However, it is noteworthy that all of the patients with glecaprevir-induced hyperbilirubinemia had chronic hepatitis but not cirrhosis. Thus, it is not necessarily safe for patients with chronic hepatitis to undergo the glecaprevir/pibrentasvir regimen regarding development of hyperbilirubinemia. Glecaprevir is a inhibitor of OATP1B1 and OATP1B3^7^, which are primarily responsible for the hepatic uptake of unconjugated bilirubin^5^. Because glecaprevir-induced hyperbilirubinemia did not necessarily occur at a low frequency in the present study, caution should be exercised in the development of hyperbilirubinemia by drug-bilirubin interaction. Considering the findings that all of the patients who developed hyperbilirubinemia during glecaprevir/pibrentasvir treatment had chronic hepatitis, the main factor might be functional fluctuation of transporter.

Several studies revealed that gadoxetic acid-enhanced MR imaging is a promising tool in the quantification of liver fibrosis and hepatic function^17–20^. Thus, we utilized MR imaging before treatment to estimate the intra-hepatocyte accumulation of glecaprevir through quantitative analysis of parenchymal enhancement at the hepatobiliary phase. Predictably, we found that the enhancement effect significantly and independently correlated with glecaprevir C_trough_. It can result from the properties of gadoxetic acid, which is specifically taken up into hepatocytes by uptake transporters such as OATP1B1 and OATP1B3^14^. Hepatic impairment, drug–drug interactions, and genetic polymorphisms can affect the function of OATPs in patients with chronic liver disease^21,22^. Similarly, enhancement of liver parenchyma by gadoxetic acid may be influenced not only by hepatic impairment but by polymorphisms in the genes encoding OATPs^21,23^. In particular, our previous study precisely demonstrated that, independent of hepatic impairment, the SLCO1B1*1B haplotype and *SLCO1B3* 334T > G polymorphism affected the liver parenchymal enhancement of gadoxetic acid^21^. Unconjugated bilirubin is taken into hepatocytes via several OATPs^5,24^. Similarly, glecaprevir was also shown to be a substrate of OATP1B1 and OATP1B3^7^. For all these reasons, gadoxetic acid-enhanced MR imaging may indicate decreased expression of OATPs resulting from hepatic impairment and interindividual variability in OATP function by single nucleotide polymorphisms. Therefore, the gadoxetic acid-enhanced MR image could indirectly reflect glecaprevir exposure and predict the development of glecaprevir-induced hyperbilirubinemia. In this study, 5 of 33 patients (15%) had elevated total bilirubin above CTCAE grade 2, which is greater than the previously reported frequency^12^. The difference in the development of grade 2 hyperbilirubinemia could result from the cohort size between our small cohort (n = 33) and the cohort of real-world data in Taiwan^12^ (n = 742). In particular, it may be explained by the difference in the allele frequencies of *SLCO1B1*.

Recently, it has been shown that the liver enhancement effect at the hepatobiliary phase of gadoxetic acid-enhanced MR imaging correlates with the C_trough_ of paritaprevir, a NS3/4A protease inhibitor, and could predict the development of hyperbilirubinemia with an incident rate of 27% during an ombitasvir/paritaprevir/ritonavir regimen^10^. Although the incidence rate of hyperbilirubinemia has occurred with less frequency at 15% during the glecaprevir/pibrentasvir regimen, we clearly indicate in the present study that the enhancement effect of gadoxetic acid MR imaging is useful for prediction of glecaprevir C_trough_ and the incidence of hyperbilirubinemia. It should be emphasized that the odds ratio of the occurrence of hyperbilirubinemia for those with CEI ≤ 1.71 relative to those with > 1.71 was approximately 14.0. These findings lend support to the efficacy of gadoxetic acid-enhanced MR imaging on prediction of hyperbilirubinemia. Additionally, our analysis suggests that the enhancement effect of gadoxetic acid-enhanced MR imaging was associated with the degree of hyperbilirubinemia, especially in maximum indirect bilirubin concentration. According to our results, pretreatment MR imaging with gadoxetic acid prior to the anti-viral therapy can not only visualize hepatocellular carcinoma but also define high risk patients for development of hyperbilirubinemia.

The ROC curve and multivariate analyses indicated that the best cut-off value for CEI at 20 min was 1.71, and that CEI was an independent pretreatment factor for predicting glecaprevir-induced hyperbilirubinemia. These findings enabled discrimination of HCV patients undergoing the glecaprevir/pibrentasvir regimen at risk of developing hyperbilirubinemia. CEI also correlated independently with the C_trough_ of glecaprevir, which could explain the fact that the hepatobiliary phase of gadoxetic acid-enhanced MR imaging reflects OATP function. However, an important problem in the image analysis is the accuracy of the enhancement effect of MR images. First, optimal measuring methods for estimating OATP function have not been established^25^. Second is the difficulty in estimating OATP function using gadoxetic acid-enhanced MR imaging. As a matter of fact, biliary excretion has already occurred at the hepatobiliary phase at 20 min. To assess the OATP function, it would be desirable to take several images after administration of the contrast agent and to assess the enhancement change over time^26^.

To our knowledge, this is the first study to examine the association between quantitative analysis of hepatic enhancement by gadoxetic acid-enhanced MR imaging and prediction of glecaprevir-induced hyperbilirubinemia. However, gadoxetic acid-enhanced MR imaging could not necessarily be performed in all patients for an institutional convenience in the clinical settings. Since the indirect bilirubin value also has a high discriminative ability to predict grade ≥ 2 hyperbilirubinemia, it may be reasonable that only patients with an indirect bilirubin concentration ≥ 0.5 mg/dL, best cutoff value, should undertake MR imaging at least in these circumstances. In the future, this study could be of practical use in predicting the response to plasma exposure to an anticancer drug that is a substrate for OATP1B1/1B3. Furthermore, the imaging analysis could be used to identify adverse events related to the elevation of plasma concentration of these drugs.

Our study has a few limitations. First, the number of subjects in this study was small, enrolled from only two facilities. Second, the number of cirrhotic patients represented in the current study is small. Third, we have examined only C_trough_ of glecaprevir. Additional studies in larger numbers of patients are needed to identify not only the C_trough_ but the area under the plasma concentration curve for glecaprevir after dosing. Despite these limitations, it should be noted that MR imaging could be a promising imaging parameter to visualize pharmacokinetics and pharmacodynamics of glecaprevir during treatment.

In conclusion, this study showed that liver parenchymal enhancement in gadoxetic acid-enhanced MR imaging performed at pretreatment was associated with the concentration of glecaprevir. In addition, hypo-enhancement effect on the hepatobiliary phase was an independent predictor for development of hyperbilirubinemia. Patients whose gadoxetic acid-enhanced MR imaging indicates hypo-enhancement would require special monitoring for hyperbilirubinemia with precise examination, including the value of serum indirect bilirubin.

## Methods

### Patients and treatments

Our study was a two-facility, exploratory, prospective study. The study enrolled adult patients with HCV genotype 1b, 2a or 2b chronic hepatitis or compensated liver cirrhosis using the glecaprevir/pibrentasvir combination (Maviret^®^, Abbvie, Tokyo, Japan) at both Juntendo University Nerima Hospital (Tokyo, Japan) and Nippon Medical School Chiba Hokusoh Hospital (chiba, Japan) between Nov 2018 and June 2021.

The study was approved by the ethics boards of both Juntendo University Nerima Hospital (No. 18-20) and Nippon Medical School Chiba Hokusoh Hospital (No. 530010) and was carried out in accordance with the 1964 Declaration of Helsinki and its later amendments. The study plan was also registered as UMIN000034251. Written informed consent was obtained from each patient before enrollment. Patients with renal impairment (estimated glomerular filtration rate < 40 mL/min/1.73 m^2^) were excluded. Patients taking atorvastatin or ursodeoxycholic acid, a substrate of OATP1B1 and OATP1B3, were excluded, and there were no patients taking substrates or an inhibitors of OATP1B1 and OATP1B3 (e.g., other statins, fexofenadine, clarithromycin, and rifampicin) other than atorvastatin and ursodeoxycholic acid.

We decided to sample data in the study in terms of the relationship between the C_trough_ of glecaprevir and the CEI. Because a previous report showed that the correlation coefficient between the C_trough_ of paritaprevir and the CEI was 0.612^10^, we assumed that the correlation coefficient of the study regarding glecaprevir was 0.6. Sample sizes were calculated to determine the number of patients required for a significant correlation coefficient between the C_trough_ of glecaprevir and the CEI. The number of patients required to meet our significance level (5%) and margin of error (< 20%) was 20. We ultimately decided to collect data from at least 30 patients. This study involved 33 Japanese patients (15 males and 18 females), with ages ranging from 22 to 91 years. Twenty-eight patients with chronic hepatitis and five with liver cirrhosis of Child–Pugh A were included. All patients were treated with glecaprevir/pibrentasvir at a dose of 300/120 mg, administered orally once daily for 8 or 12 weeks. The duration of anti-HCV therapy was determined according to the indication criteria approved by the Japanese government. For patients with liver cirrhosis or treatment failure with past DAA therapy, the total treatment duration was 12 weeks.

Gadoxetic acid-enhanced MR imaging was performed and biochemical examination of blood was obtained within one month prior to starting glecaprevir/pibrentasvir treatment. The FIB-4 index, a serological marker of liver fibrosis, was also calculated before treatment. Serum total and indirect (unconjugated) bilirubin concentrations were monitored during antiviral therapy, and toxicities related to hyperbilirubinemia were graded according to the CTCAE, version 4.0. Hyperbilirubinemia in this study was defined as CTCAE grade ≥ 2. Because T_1/2_ of glecaprevir is 5.7–9.9 h^4^, the steady-state plasma C_trough_ of glecaprevir was determined from blood samples obtained 7 days after the start of treatment. Blood samples were centrifuged within 30 min at 1500 rpm for 10 min at 4 °C, and plasma concentrations of glecaprevir were determined using liquid chromatography-tandem mass spectrometry, as previously described^27^.

### MR imaging analysis

Gadoxetic acid-enhanced magnetic resonance MR images were obtained with either a 1.5-T (Signa Exite; GE Medical Systems, Milwaukee, WI) or a 3.0-T MR (Discovery MR750 3.0 T; GE Medical Systems) system. The imaging protocols consisted of T1-weighted, fat-suppressed 3D gradient-recalled echo sequences using parallel imaging with phased-array uniformity enhancement. After obtaining pre-contrast scans, gadoxetic acid (0.1 mL/kg body weight; EOB Primovist™ injection; Bayer Schering Pharma, Berlin, Germany) was given intravenously, as a bolus at a rate of 3 mL/s. Parameters for the 1.5-T system were as follows: repetition time 4.0 ms; echo time 1.9 ms; flip angle 15°; matrix 352 × 224; field of view 400 × 360 mm; acquisition time 19 s; slice thickness, 2.2 mm. The parameters for the 3.0-T system were as follows: repetition time 4.2 ms; echo time 1.9 ms; flip angle 15°; matrix 320 × 192; field of view 340 × 340 mm; acquisition time 10 s; slice thickness, 2.5 mm. Hepatobiliary images at 20 min after administration were pictured in the transverse plane and quantified using a contrast enhancement index (CEI)^17,28^, with the number of measurement regions modified^29^. To put it simply, pre-contrast and hepatobiliary images were displayed with FuncTool™ software (GE Medical Systems), and 100–150 mm^2^ regions of interest (ROIs) of the bilateral major psoas muscles and 12 points of the liver excluding hepatic cysts, large vessels and prominent artifacts were selected. ROIs were identified by a combination of Couinaud’s segments (S1–S8) and zonal locations (central or peripheral) as follows: S1—central, S2—central, S2—peripheral, S3—central, S3—peripheral, S4—central, S4—peripheral, S5—central, S6/7—central, S6—peripheral, S7—peripheral, and S8—peripheral^9^. The liver to major psoas muscle ratios (LMRs), based on average of left and right signal intensities (SIs), were calculated before (SI_pre_) and at 20 min after (SI_20_) gadoxetic acid administration. ROIs of the same size and shape were placed at the same points on transverse images, and CEI were calculated as LMR_20_/LMR_pre_. Blind imaging analyses were made by two radiologists who were not authors, and each had more than 10 years of experience in MR imaging. The average value of all SIs measured by the two radiologists were adopted for analysis of the CEI. They did not know the laboratory data and clinical course. The interval between readout sessions was 2–10 months after MR imaging. The average value of each SI determined by two radiologists was adopted for analysis.

### Statistical analysis

Continuous variables are expressed as median values (range). Statistical analyses were performed using Fischer’s exact test, or Mann–Whitney’s U test. Pearson’s correlations were performed to determine the associations between pairs of variables, after the data were tested for normal distribution by the Shapiro–Wilk normality test. Variables of pretreatment factors with *p* values < 0.20 in the univariate analysis were re-evaluated by multiple logistic regression analysis using the forward selection method to identify the factors associated with the development of hyperbilirubinemia. All tests were two-sided and *p* values < 0.05 were considered to be statistically significant. These statistical analyses were performed using SPSS Statistics for Windows, Version 22 (IBM Corp., Tokyo, Japan).

## Data Availability

The datasets that support the findings of this study are available from the corresponding author upon reasonable request.
